# Retraction: A synthetic peptide designed to neutralize lipopolysaccharides attenuates metaflammation and diet-induced metabolic derangements in mice

**DOI:** 10.3389/fimmu.2025.1561887

**Published:** 2025-01-28

**Authors:** Frontiers Editorial Office

Following publication, concerns were raised regarding the integrity of the images in the published figures. Specifically, unexpected similarity was detected between western blot images presented in Figure 7. An investigation was conducted in accordance with Frontiers’ policies. The authors provided raw data for assessment, but the concerns could not be fully resolved. As a result, the data and conclusions of the article have been deemed unreliable and the article has been retracted.

This retraction was approved by the Chief Editors of Frontiers in Immunology and the Chief Executive Editor of Frontiers. The authors disagree with the retraction.

